# Matrix Effectors and Cancer

**DOI:** 10.3390/cancers14010200

**Published:** 2021-12-31

**Authors:** Zoi Piperigkou, Nikos K. Karamanos

**Affiliations:** 1 Biochemistry, Biochemical Analysis and Matrix Pathobiology Research Group, Laboratory of Biochemistry, Department of Chemistry, University of Patras, 265 04 Patras, Greece; 2 Foundation for Research and Technology-Hellas (FORTH)/Institute of Chemical Engineering Sciences (ICE-HT), 265 04 Patras, Greece

Extracellular matrices (ECMs) are highly dynamic three-dimensional structural meshworks composed of macromolecules, such as proteoglycans/glycosaminoglycans (PGs/GAGs), collagens, laminins, elastin, (glyco)proteins, and matrix-degrading enzymes, such as proteases and glycosidases [1]. Matrix macromolecules are characterized by high structural complexity and heterogeneity. They form complex networks through which they dynamically communicate with cells, thus serving as critical regulators of several homeostatic and pathological processes, such as cancer. ECM molecular composition varies by the tissue of origin, and it undergoes significant remodeling during cancer progression. The elucidation of the mechanistic aspects governing matrix assembly and cell–matrix interactions is of critical importance for a deeper understanding of matrix-mediated cancer pathobiology and to discover novel therapeutic approaches. In this Special Issue entitled, “Matrix Effectors and Cancer”, several original research articles and comprehensive reviews are included to highlight the emerging roles of effective matrix macromolecules, including PGs/GAGs, matrix-remodeling enzymes, membrane receptors, specific types of collagens, matrix (glyco)proteins and genomic alterations that play key roles in the development of several malignancies, as well as in paracrine interactions among cancer cells and tumor stroma that modulate cancer cell aggressiveness.

Differential PG gene expression and GAG structural modifications are closely related to the availability of growth factors as well as cancer cell signaling [2]. Sulfated GAGs serve as promising pharmacological targets, acting on different steps of cancer metastasis. Silva et al. reported that the heparan sulfate (HS) isolated from the viscera of the ascidian *Phallusia nigra* has very low anticoagulant and antithrombotic activities and a reduced hypotension potential. Notably, this GAG drastically attenuates the metastatic potential of colon carcinoma cells in vivo [3]. Another novel study of this Special Issue correlated the filopodia formation with hyaluronan (HA) synthesis in a quantitative analysis. Kyykallio et al. addressed for the first time the direct quantification of filopodial traits, measuring the length and density of these protrusions in a series of human cancer cell lines with variable levels of HA synthesis. The results of this work reveal that the abundance and length of filopodia in cancer cells is associated with the activity of HA synthesis in a CD44-independent manner [4]. Nassar et al. analyzed the function of the cell surface PG, syndecan-1, in tumor angiogenesis in a 3D human umbilical vein endothelial cell (HUVEC) co-culture system. They found that syndecan-1 released from triple-negative breast cancer cells controls angiogenesis in a tissue factor (TF)-related mechanism and the prognosis of breast cancer patients [5]. The functional role of syndecan-1 has also been highlighted by Reszegi et al., who demonstrated the implication of syndecan-1 in lipid metabolism serving a protective role in hepatocarcinogenesis through the inhibition of the mTOR and β-catenin pathways [6].

This Special Issue also includes studies on the identification of novel prognostic and therapeutic biomarkers implicated in cancer pathobiology. Pinto et al. correlated the expression of the extracellular PG, biglycan, with tumorigenic gene signatures and poor patient prognosis in advanced stages of gastric cancer [7]. Moreover, Magnussen et al. critically reviewed up-to-date studies on nephronectin, analyzed the structure and domain-related functions of this ECM protein and linked these functions to potential roles in cancer progression [8]. Nallanthighal et al. discussed the functional role of collagen type XI alpha 1 (COL11A1) as a promising cancer biomarker [9].

The fundamental role of membrane receptors has long been studied to unravel the regulatory mechanisms of matrix–cancer cell interactions to further promote cancer progression. Tzanakakis et al. discussed key studies focusing on insulin-like growth factor (IGF) downstream signaling in bone sarcomas (i.e., osteosarcoma, chondrosarcoma and Ewing sarcoma), focusing on the dynamic interplay among IGF, tumor microenvironment (TME) constituents and matrix effectors [10]. Jeanne et al. concluded an advanced preclinical characterization of TAX2 peptide, a cyclic peptide acting as an orthosteric antagonist for thrombospondin-1 (TSP-1) interaction with the receptor CD47. Using relevant syngeneic ovarian carcinoma models, they highlighted the ability of TAX2 to convert poorly immunogenic tumors into ones displaying effective antitumor T cell immunity [11]. Kolliopoulos et al. demonstrated that the cleavage of HA receptor, CD44, in A549 lung cancer cells and other cells is promoted by transforming growth factor-beta (TGF) in a manner that is dependent on ubiquitin ligase tumor necrosis factor receptor-associated factor 4 or 6 (TRAF4 or TRAF6, respectively) and that TRAF4/6 mediates the pro-tumorigenic effects of CD44 [12]. Another interesting work in this Special Issue focuses on the role of discoidin domain receptors (DDRs) in tumor development and metastasis. Majo and Auguste described in their comprehensive review the emerging DDR inhibitory strategies, which could be used as new alternatives for cancer therapeutic approaches [13].

Cancer progression is also governed by the action of matrix-remodeling enzymes; thus, ECM enzymatic activity has long been the focus of oncology research. Piperigkou et al. advocated a comprehensive review of the types of major matrix-remodeling enzymes, including matrix metalloproteinases (MMPs), plasminogen activation system components, cathepsins and glycolytic enzymes (i.e., heparanase and hyaluronidases). They highlighted the effects of their enzymatic functions in cancer initiation, propagation and progression as well as their pharmacological targeting, and ongoing clinical trials are critically discussed [14]. Vallet et al., using in vitro binding assays and computational tools, analyzed for the first time the interactome of the five members of the lysyl oxidase (LOX) family to verify its molecular and biological functions, and the signaling pathways mediating these functions. The results of this study demonstrate that LOXL2 builds a large interactome network participating in cell–ECM interactions mediated by non-integrin and integrin receptors, protein folding and chaperone activity, organ and blood vessel development, cellular response to stress and signal transduction in colorectal carcinoma [15]. Dauvé et al. characterized the interactions of MMP-14 with the small leucine-rich PG (SLRP) lumican-derived peptides that inhibited MMP-14 proteolytic activity and the aggressive properties of melanoma cells [16].

Post-translational modifications (PTMs) are critical for the functions of many proteins, both intracellular and in the matrisome. The definition of the human matrisome, as the ensemble of matrix-encoded genes and ECM-affiliated and -secreted proteins, degrading and cross-linking enzymes, is critical for the analysis of matrix organization and functions in (physio)pathological processes [1]. Holstein et al. studied a large Pan-Cancer cohort spanning 32 tumor types and demonstrate the specificities of matrisome PTM-affecting mutations over the rest of the genome, also evidencing features and findings that might be relevant for prognostication and mechanistic understanding of the supportive role of the TME in the tumorigenic process [17]. In another interesting study of this Special Issue, Izzi et al. investigated the genomic alterations of matrisome genes in several cancer types and their consequences. Mining The Cancer Genome Atlas (TCGA) data, they found that alterations and mutations in matrisome genes are predicted to significantly affect gene expression and protein function and highlighted that these studies will further improve our understanding of the roles of the matrisome in targeting the ECM during cancer progression [18]. Zolota et al., in their comprehensive review, highlight major epigenetic alterations (i.e., DNA methylation, chromatin remodeling and non-coding RNAs) of essential matrix components and epithelial-to-mesenchymal transition (EMT) in triple-negative breast cancer in an effort to provide perspectives for the future design and implementation of diagnostic and therapeutic suggestions [19].

The multistep process of metastatic potential is evoked by the coordinated interactions among cancer cells and the tumor stroma, where the matrix reorganization boosted by the recruited factors (i.e., secreted growth factors, cytokines, PGs/GAGs and deposited ECM fragments by enzymatic activity) is critical to form the pre-metastatic niches [20]. Nikolopoulou et al., in their comprehensive review, highlight the vital role of cell adhesion in malignancy and describe how adhesion components regulate tumor stroma responses and control cancer development. The role of the adhesome as a signaling and mechanosensing hub, orchestrating cellular responses that shape the tumor milieu, has also been described [21]. Javadi et al. demonstrated that syndecan-1 overexpression affects the angiogenic factor secretion of mesothelioma cells and thereby inhibits endothelial cells proliferation, tube formation and wound healing in a VEGF-dependent manner [22]. Mavrogonatou et al. revealed that stromal senescence, as a side-effect of radiotherapy, results in the tumor-promoting phenotypic trait of ionizing radiation-induced prematurely senescent human stromal fibroblasts, which is correlated with the decreased deposition in the pericellular matrix of the SLRP, decorin. Senescence-associated decorin downregulation is mediated by bFGF and VEGF in an autocrine manner, while autophagy activation through mTOR inhibition enhanced decorin expression [23]. Finally, a compelling study in this Special Issue was conducted by Caon et al., who identified a novel target to induce HA synthase 2 (HAS2) in stromal fibroblasts. Importantly, a yet uncharacterized protein secreted by breast tumor cell lines, named c10orf118, is positively correlated with breast cancer patient survival and low metastasis [24].

Novel mechanistic aspects governing matrix-mediated cancer progression are presented and summarized in the original research articles and up-to-date critical reviews comprising this Special Issue, entitled “Matrix Effectors and Cancer”. This in-depth understanding of matrix assembly complexity during cancer development may continue to drive improvements in theranostics focusing on patient-centered care.
